# MDR1 Gene Polymorphisms and Its Association With Expression as a Clinical Relevance in Terms of Response to Chemotherapy and Prognosis in Ovarian Cancer

**DOI:** 10.3389/fgene.2020.00516

**Published:** 2020-05-26

**Authors:** Absarul Haque, Khalid Hussain Wali Sait, Qamre Alam, Mohammad Zubair Alam, Nisreen Anfinan, Abdul Wahab Noor Wali, Mahmood Rasool

**Affiliations:** ^1^King Fahd Medical Research Center, Faculty of Applied Medical Sciences, King Abdulaziz University, Jeddah, Saudi Arabia; ^2^Department of Medical Laboratory Technology, Faculty of Applied Medical Sciences, King Abdulaziz University, Jeddah, Saudi Arabia; ^3^Gynecology Oncology Unit, Department of Obstetrics and Gynecology, Faculty of Medicine, King Abdulaziz University Hospital, Jeddah, Saudi Arabia; ^4^Center of Excellence in Genomic Medicine Research, Faculty of Applied Medical Sciences, King Abdulaziz University, Jeddah, Saudi Arabia

**Keywords:** multidrug resistance, MDR1 gene, C3435T polymorphism, ovarian cancer, chemotherapeutic response

## Abstract

In spite of the significant advancements in the treatment modalities, 30% of advanced stage ovarian cancer (OC) patients do not respond to the standard chemotherapeutic regimen and most of the responders finally relapse over time due to the escalation of multidrug resistance (MDR) Phenomenon. Our present study evaluated chemotherapeutic sensitivity response among 47 ovarian tumor patients of which we found 37 (78.8%) sensitive and remaining 10 (21.2%) resistant. Among the resistant, seven tumor samples were found to be platinum resistant or refractory to platinum (CB/TX), one to carboplatin, and two to 5FU. Notably, all these resistant cases were observed in the disease recurrence group of patients identified at stage III or IV. The stage III resistant cases revealed heterozygous mutation (C/T) in exon 12 (C1236T) and 26 (C3435T) and increased level of mRNA, whereas homozygous mutation (T/T) was found at stage IV tumor patients. The genotypic difference was found to be significant (*p* = 0.03) for exon 12, and *p* = 0.003 for exon 26 mutant genotypes. No significant association between genotypes of different exons with tumor stages and tumor grade was observed (*p* > 0.05). However, a significant association was observed between the genotype of exon-12 and histopathology of tumor tissue (*p* = 0.028). Statistically, the chemotherapy response was found to be significantly associated with the tumor stage (*p* = 0.019). We also observed a significant difference in PFS (*P* = 0.019) and OS (*P* = 0.047) between tumor grades 1 and 3. Notably, the highest mRNA expression was observed in resistant tumor sample T-32, where interestingly we found homozygosity TT in all of the exons 12, 21, and 26. Thus, we suggest that exons 12 (C1236T) and exon 26 (C3435T) polymorphism may play a role in inducing drug resistance by altering the expression level of the MDR1 gene. To summarize, we suggest that the expression of MDR1 in OC is influenced by tumor stage and genotype variants as well as by chemotherapeutic drugs. Thus our findings suggest that inter individual variability in platinum based therapy may be anticipated by MDR1 genotypes. Further studies on a large number of samples shall eventually lead to provide beneficial information for the individualized chemotherapy.

## Introduction

Ovarian cancer (OC) represents the fifth leading cause of death among women, but ranked number one in deaths due to gynecological malignancies (Cancer Facts and Figures, 2018; Torre et al., 2018). The ovarian tumors are classified into three major groups: epithelial/stromal, germ cell, or sex cord/stromal (Horta and Cunha, 2015). The vast majority (90%) represents Epithelial Ovarian Cancer (EOC), largely of serous type, but endometrioid and clear cells as well as mucinous variants are also present (Siegel et al., 2016; Torre et al., 2018). In spite of the advancement in diagnostic and therapeutic procedures, EOC is still the major cause of high morbidity and mortality among all female reproductive malignancies due to the late stage manifestation of the disease (Jemal et al., 2011; Bowtell et al., 2015; Siegel et al., 2016). Notably, the high death rate, i.e., 5% accounts for EOC as compared to its low representation as only 2.5% of all female cancer cases are due to the fact that four out of five EOC patients are diagnosed at advanced stages of the tumor progression, i.e., when it spreads all over the abdominal cavity (Torre et al., 2018). Regardless of the clinical stage, the standard management of EOC includes cytoreductive surgery followed by platinum (Cisplatin or Carboplatin) based on chemotherapy, i.e., a combination of Carboplatin or Cisplatin with the Taxol, such as paclitaxel drugs (Wright et al., 2015, 2016; Trimbos, 2017). Notably, the present standard chemotherapy regimens apply for the treatment of OC consist of a combination of Carboplatin and Paclitaxel or either as an alternative approach comprised of a combination of Carboplatin and Gemcitabine (Trope and Kaern, 2006; Wright et al., 2015). In spite of these possible approaches, most of the patients have been identified with relapsed disease. It has been estimated that around 20% of the patients do not respond to the platinum anti cancer drugs (Platinum-refractory) and such cases are considered as early relapses, since the disease occurs within the first 6 months of post therapy (Trope and Kaern, 2006). However, the remaining 80% are termed as a Platinum-sensitive as those cases show relapse later. Thus, in Platinum-sensitive relapse patients usually a combination of Carboplatin/Paclitaxel is the foremost chemotherapeutic choice as a palliative value in order to achieve the slow progression of the disease, reduction in pain, and managing the better quality of life (Cannistra, 2004). Despite the fact that many chemotherapeutic drugs are available for ovarian cancer, none is fully effective in curbing tumor growth owing to the emergence of multidrug resistance (MDR) in the tumor cell population (Ren et al., 2016; Guo et al., 2018). Multiple research findings have reported the involvement of two molecular “efflux pumps” namely *P*-glycoprotein (*P*-gp) and multidrug resistance associated protein (MRP) in tumor cell membrane, that are often responsible for MDR phenomenon in cancer cells by effluxing out the therapeutic agents from the cell (Kartal-Yandim et al., 2016; Ren et al., 2016). Among them, the *P*-gp which is foremost characterized and widely investigated for its implication in MDR, belongs to ATP– binding cassette (ABC) of the transporter, encoded by ABCB1/MDR1 gene (Gottesman, 2002; Żesławska et al., 2016). The *P*-gp transporter is able to actively efflux out approximately 20 cytostatic drugs from the cell, including paclitaxel, doxorubicin, and vincristine (Dean et al., 2001; Kelland, 2007; Podolski-Renić et al., 2011). Several *in vitro* and *in vivo* studies have confirmed that P-gp/MDR1expression is the highest in tumor derived tissues as compared to normal tissues and also as multidrug resistant cancer cells which produce larger extracellular vesicles (EVs) than their sensitive cellular counterparts (Baekelandt et al., 2000; Yusuf et al., 2003; Lopes-Rodrigues et al., 2016). Further studies revealed that the *P*-gp/MDR1 expression level is up regulated in cancer cell in response to the chemotherapeutic agents and the deletion of *P*-gp (MDR1 knockout mice) display significant enhancements in the accumulation of *P*-gp substrates within the cell (Goldstein et al., 1989; Noonan et al., 1990; Gottesman, 2002). Recently, *in vitro* study has demonstrated the enhanced expression of *P*-gp in both transcript and protein levels in ovarian and other cancer derived cell lines which contribute resistance to paclitaxel and other anticancer drugs (Januchowski et al., 2013; Kim et al., 2017). Notably, Multiple mutational analysis revealed that MDR1 is genetically quite variable, suggesting that single nucleotide polymorphisms (SNPs), may have significant effects on the expression and function of *P*-gp transporter (Potocnik et al., 2001; Meissner et al., 2004; Sakaeda et al., 2004; Li et al., 2006; Potocnik et al., 2008). In this effort, many researchers have analyzed that the SNPs within the gene of the MDR1/*P*-gp transporter, have established a relationship with variation in expression and function of MDR1, which eventually lead to the affect responsiveness to drugs, along with susceptibility to disease (Kelland, 2007; Sharom, 2008; Tsai et al., 2015). At least 50 SNPs have been identified within MDR1 gene positions on chromosome 7, and the 3843 bp coding region comprises 28 exons (Sauna et al., 2007; Vine et al., 2014). Most importantly, among SNPs reported in the MDR1 gene, three insertion/deletion SNPs, C1236T in exon 12, G2677T/A in exon 21 and C3435T located in exon 26 respectively, have been most widely investigated and were determined to be functionally significant and ethnically found to be different when mapped at this region of gene (Kelland, 2007; Sharom, 2008; Miyata et al., 2016). Further, Hoffmeyer et al. (2000) findings suggested that synonymous SNP C3435T in exon 26 plays an important role in the function of *P*-glycoprotein. Many studies have established that linkage disequilibrium of C3435T with two common SNPs, C1236T (exon 12) a synonymous, and tri-allelic G2677T/A (exon 21) a non-synonymous SNPs, respectively. The SNPs C3435T in exon 26 (Ile1145Ile) is associated with reduced MDR1 mRNA expression (Tang et al., 2002; Sai et al., 2003; Yin et al., 2009). Notably, Gréen et al. (2006) showed that a non-synonymous (Changed in amino acid) SNPs G2677T/A having both alleles homozygous are better in responding to the treatment with paclitaxel compared to those groups of ovarian cancer patients having at least one wild type allele in. Hence, it is indicated that *P*-gp plays a pivotal role in conferring paclitaxel response in ovarian cancer in terms of drug response and susceptibility and may provide useful information for personalized therapy (Gréen et al., 2006). Thus, further determination of genetic variations in MDR1 gene in a particular population may be vital for individualized pharmacotherapy.

Knowing the important role of MDR1 in predicting treatment response with chemotherapeutic drugs, we evaluated the SNPs variants in C1236T in exon 12, G2677T/A in exon 21 and C3435T in exon 26, and compared its association on mRNA expressions in tumor tissues in order to establish their relevance in terms of the response to chemotherapy and prognosis in Saudi ovarian cancer women.

## Materials and Methods

Fifty-two patients with primary ovarian cancer and 19 women as a control group were enrolled in this study. The controls were recruited in the same age group as the patient so that there were no significant differences regarding the age and sex between cases and control groups Freshly collected EOC tumor samples from each patient and blood from each healthy individual were transported to the lab in refrigerated condition. The study was approved by the local Ethical Committee Board, King Abdulaziz University. Ethical clearance was also taken from King Fahd Medical Research Center, KAU. The written informed consent was obtained from all patients and control subjects according to Helsinki’s declaration. The EOC tumor tissue specimens were collected from the Gynecology Oncology Unit, Obstetrics and Gynecology Department, Faculty of Medicine, King Abdulaziz University Hospital, Jeddah between November 2015 and January 2018. Histological diagnosis was confirmed for all samples included in this study. In this study, 52 OC patients were selected for SNPs and mRNA analysis of MDR1 gene, but 47 cases were evaluated for chemotherapeutic response since, five patients namely (T-15, T-29, T-39, T-48, and T-52) did not turn-up in follow up studies due to severely sick or refused to get the treatment. Therefore, we excluded those five samples as their chemotherapeutic response and relapse cases data were not available for the chemo sensitivity assay.

### RNA Extraction From Fresh Tumor

Total RNA was isolated from freshly collected ovarian tumor tissues using the Animal tissue RNA purification kit (Norgen, Biotek Corporation Catalogue # 25700) following the manufacturer’s protocol. The total RNA quantity and quality were quantified and assessed using the Nano Drop (Thermo Scientific, United States).

### RNA Isolation From FFPE Tumor Tissue

Total RNA was extracted from Formalin-Fixed paraffin-Embedded (FFPE) ovarian tumor tissue using PureLink^TM^ FFPE RNA Isolation Kit (Kit No. 156002), Thermo Fisher Scientific, United States, following the manufacturer’s protocol. The total RNA quantity and quality were quantified and assessed using the Nano Drop (Thermo Scientific, United States).

### Reverse Transcription Polymerase Chain Reaction (RT-PCR)

In order to check the mRNA expression of MDR1, complementary cDNA was reverse transcribed with total RNA using Maxima First Strand cDNA Synthesis Kit for RT-qPCR (K1641, Thermo Fisher Scientific) following the manufacturer’s instructions. In brief, 1 μg of RNA of each sample was used for cDNA synthesis. The cDNA was quantified using the Nano Drop (Thermo Scientific, United States).

### Quantitative Real-Time Polymerase Chain Reaction (qPCR)

The transcript level of the MDR1 gene was quantified by real time PCR (qPCR). An equal amount of cDNA was amplified in triplicate in 48 well optical plates (Applied Bio system) by using QuantiTect SYBER Green PCR kit (QIAGEN, Germany). In brief, 5 pmol gene specific primers (forward and reverse) as shown in Table 1 of each indicated genes were carried out in 10 μl final volume. The cDNA generated with each tumor samples, RNA was quantified separately with MDR1 primers along with two ovarian cancer cell lines (Gao et al., 2014) resistant to platinum (SK-OV-3 and OVCAR-3) were also separately analyzed in order to use one of them as a calibrator to compare the gene expression data. The endogenous control Glyceraldehyde-3-phosphate dehydrogenase (GAPDPH) was used in each tumor sample to normalize the expression against the calibrator in triplicate reaction. Step One (Applied Biosystems, United States) was used for detecting real-time PCR products. The PCR cycling conditions were chosen according to the manufactures instruction. The relative expressions were calculated by the (delta–delta) Ct method. The results (Ct) of the tested samples were normalized to GAPDH and ΔCt, which were determined as the geometric mean of the expression values against control and calibrator as described by Januchowski et al. (2013).

**TABLE 1:** Showing real time PCR (qPCR) primer sequences used for the transcript analysis.

Transcript	Sequence (5′-3′)	ENST number	Product size
MDR1 (ABCB1)	TGACAGCTACAGCACGGAAG TCTTCACCTCCAGGCTCAGT	00000265724	131 bp
GAPDH	GAAGGTGAAGGTCGGAGTCA GACAAGCTTCCCGTTCTCAG	00000229239	199 bp

### DNA Extraction From Freshly Collected Tumor and Blood Samples

DNA was isolated from 19 blood samples used as healthy controls in this study. Further, the DNA was isolated from freshly collected 19 ovarian tumor tissues following DNA extraction protocol kit (Wizard A1120, Promega, United Kingdom) utilized for both tumor and blood samples. The quality of genomic DNA was assessed by 0.8% agarose gel electrophoresis and the quantities of individual DNA preparation were determined by Nano drop.

### DNA Isolation From FFPE Tumor Tissue

Remaining DNA was isolated from 33 FFPE ovarian tumor tissues using PureLink^TM^ FFPE DNA Isolation Kit (Kit No. 0881) (Thermo Fisher Scientific, United States) following the manufacturer’s protocol. The total DNA quantity and quality were quantified using the Nano Drop (Thermo Scientific, United States).

### PCR Amplification of MDR1 Gene

The exon specific primers were designed and synthesized from Macrogen Inc, South Korea (Table 2). The length of the PCR amplified product size of ABCB1 exon 12, exon 21 and exon 26, was 169, 156, and 161 bp, respectively. The PCR reaction was performed in 25 μl total volume. In brief, 1 μg of genomic DNA was used as a template to amplify with 10 pmoles exon specific primer pairs with Green master mix Taq polymerase (Promega, United Kingdom). Thermal cycle condition was as follows; initial denaturation at 95°C for 2 min, followed by cycle denaturation at 95°C for 45 s; annealing 52–56°C for 2 min (depending on the primer pairs used); extension at 72°C for 50 s for 35 cycles; followed by final extension at 72°C for 5–7 min as describe by Januchowski et al. (2013).

**TABLE 2:** Showing the specific primers pair used for ABCB1 exon amplification.

Genes	Sequence (5′-3′)	Exon	Amplicon size (bp)
MDR1 (ABCB1)	GTTCCTATATCCTGTGTCTGT TCATAGAGCCTCTGCATCAGCT	12	169
	GCAATTGTACCCATCATTGCAA ACACTGATTAGAATACTTTACT	21	156
	CATCCTGTTTGACTGCAGCAT TCCCAGGCTGTTTATTTGAAG	26	161

### RFLP Analysis of C3435T (Exon-26) Polymorphism

The 161 bp amplified exon 26 PCR product of all 52 tumor samples were subjected for Restriction Fragment Length Polymorphism (RFLP) analysis in order to determine the genotypes of C3435T (exon 26) MDR1 gene as shown in Figure 3. In brief, 500–1000 ng of the purified PCR product was digested with *Mbo*I restriction enzyme in a total 15 μl reaction volume at 37°C overnight. The generated fragment of varied length depending on the genotype of the subject were assessed by loading the digested mixture of each sample on 2% agarose gel (Owen et al., 2005). These RFLP generated genotypes produce three patterns of band at position C3435T. For example, wild type (C allele) shows two bands (89 and 72 bp), homozygous mutant (T allele) shows one band (161 bp) and heterozygous CT genotype shows as three bands (161, 89, and 71 bp), respectively as shown in Supplementary Figure P1.

### Automated DNA Sequencing Analysis of MDR1

A total of 52 samples (19 fresh tumor and 33 FFPE) of ovarian tumor obtained from different patients were subjected for genotyping exon 12 (C1236T), 21 (G2677T/A) and 26 (C3435T) of the ABCB1 gene respectively by automated DNA sequencing (Applied Biosystems 3500 XL Genetic Analyzer). In order to compare the exon sequence data between cancer and non-cancer individual, the DNA isolated from the blood of 19 healthy individual were also used to genotype the above mentioned exons. The cycle sequencing-PCR reaction was performed following the manufacture’s protocol (Big Dye terminator reaction Kit version 3.1 Applied Biosystems, United States). The sequencing primers for genotyping of exon 12 (C1236T), 21 (G2677T/A) and 26 (C3435T) of MDR1 were designed manually and also verified by using Primer3 software^1^. The list of internal primers used for cycle sequencing is shown in Table 3. The generated chromatogram of each of the exon sequenced was evaluated for the quality of sequence data by matching with standard reported sequence with the corresponding peak and SNPs were identified by analyzing the heterozygous or homozygous peak manually as shown in Supplementary Figure P2. The SNPs were further re-confirmed by comparing the heterozygous or homozygous peak in the tested DNA samples and control DNA by using nucleotide sequence analysis tools software (Finch TV). The identified SNPs were also re-confirmed by reverse strand sequencing.

**TABLE 3:** Showing internal primers used in the cycle sequencing reaction for Automated DNA sequencing of exon of the MDR1 genes.

Genes	Sequence (5′-3′)	Exon
MDR1 (ABCB1)	F- GTTCCTATATCCTGTGTCTGT	12
	F- GCAATTGTACCCATCATTGCAA	21
	F- CATCCTGTTTGACTGCAGCAT	26

### Statistical Analysis

Statistical analysis was performed using SPSS and SAS/STAT^®^ software (SAS University Edition, version 9.4M5; SAS Institute Inc. Cary NC, United States). The direct counting was carried out to calculate the allele and genotype frequencies. A difference in allele frequencies between tumor samples and normal healthy control of Saudi ethnicity was measured using student’s test. Chi-square and Fisher’s exact test were applied to detect the significance of genotype variation with tumor stage, grade and histopathology. A *p*-value < 0.05 was considered to be statistically significant. Mean expression level of mRNA of MDR1 genes between sensitive and resistant tumor samples was analyzed. Mann–Whitney, and Jonckheere–Terpstra test was performed to calculate the statistical difference in PFS and OS among various characteristics of ovarian tumor. Mann–Whitney *U* test was also performed to calculate the difference in MDR1 exon’s (wild type vs. mutant) among PFS and OS.

## Results

### Tumor Sample Characteristics

In this study, we collected a total of 52 samples of ovarian tumor in which 19 were fresh tumor and the remaining 33 were FFPE tissues. The mean age of the patients was 55.5 years. Out of these 52 samples, seven samples were categorized at stage I, four samples at stage II, thirty-five samples at stage III and the remaining six tumor samples were categorized as stage IV cancer (Table 4). The first recurrence was reported in 20 patients. Primarily, all were treated with debulking surgery and post-operative chemotherapy, which consisted of platinum based regime as well as Taxane-Platinum combination (TX/CB). However, few patients were treated with 5FU or GEMZAR and five patients namely (T-15, T-29, T-39, T-48, and T-52) did not turn-up, refused or severely sick to get the treatment. Out of 47 tumor samples, 37 (78.8%) were sensitive and the remaining 10 (21.2%) completely failed to respond to chemotherapy and were characterized as resistant having progressive disease phenotype. When we analyzed the sensitivity rate among the five chemotherapeutic agents used in this study, we found Carbo/Tax 82.5% (33/40), Carboplatin 66.7% (2/3), Fluorouracil (5FU) 33.3% (1/3) and GEMZAR 50% (1/2) sensitive respectively in OC patients. All of the resistant tumors were surprisingly found at stage III or IV. Notably, one patient (T-1) was resistant to 2 drugs (CB/TX and GEMZAR) simultaneously, so we considered T1 samples as to be resistant. The majority of the resistance was found against CB/TX (7 sample), whereas one sample was resistant to carboplatin and two to Fluorouracil (5FU) as depicted in Table 5.

**TABLE 4:** Showing patients characteristics and treatment details.

Parameter	Number
Total number of patients enrolled	52
Mean age at first diagnosis (years)	55.5
**Tumor stage**	
I	7
II	4
III	35
IV	6
**Tumor grade**	
1	14
2	7
3	31
**Histological type**	
Papillary Serous	27
Non-Serous	25
**First treatment**	
Carbo/Tax	40
Carboplatin	3
FEMARA	1
Fluorouracil (5FU)	3
Treatment refused	5
Ist recurrence (Out of 52)	20
**Second treatment**	
Carbo/Tax	7
GEMZAR	2
**Total resistant (1st and 2nd recurrence)**	
Carbo/Tax Resistant	8
Carboplatin resistant	1
Fluorouracil (5FU)	1
GEMZAR	1
Dead as a result of disease/progressive disease	21

*Clinicopathological characteristics of the EOC cases.*

**TABLE 5:** Sensitivity rates among 47 patients to five chemotherapeutic agents used in this study.

No. of Cases	Chemotherapeutic agents	Sensitive	Resistant	Sensitivity rate
40	Carbo/Tax	33	7	82.5% (33/40)
3	Carboplatin	2	1	66.7% (2/3)
3	Fluorouracil (5FU)	1	2	33.3% (1/3)
2	GEMZAR	1	1	50% (1/2)

*Out of 47 patients, 37 were sensitive and 10 patients were resistant. One patient was reported to be second line of resistant with GEMZAR.*

### MDR1 Gene Expression Analysis

**FIGURE 1:**
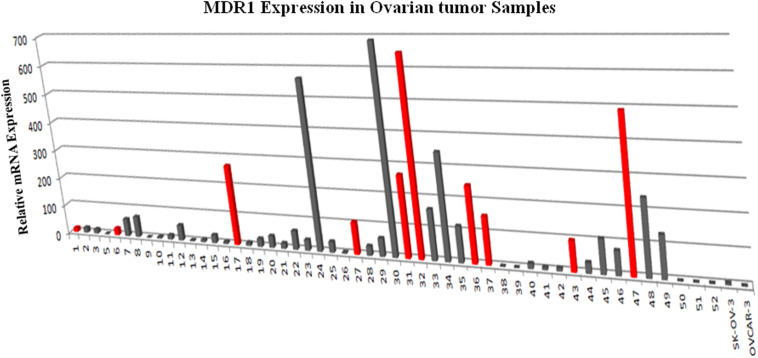
*MDR1* mRNA expression analysis by qPCR in ovarian tumor tissues tissue relative to the level expressed in OVCAR-3. The expression of *MDR1* in ovarian cancer control cell line SK-OV-3 and OVCAR-3 were measured in parallel to compare the relative expression levels between ovarian tumor tissues. The results presented were the geometric mean of values normalized to the endogenous control genes *GAPDH*. **(A)** Gray color bar depicts sensitive samples; **(B)** Red color bar depicts resistant samples respectively.

**FIGURE 2:**
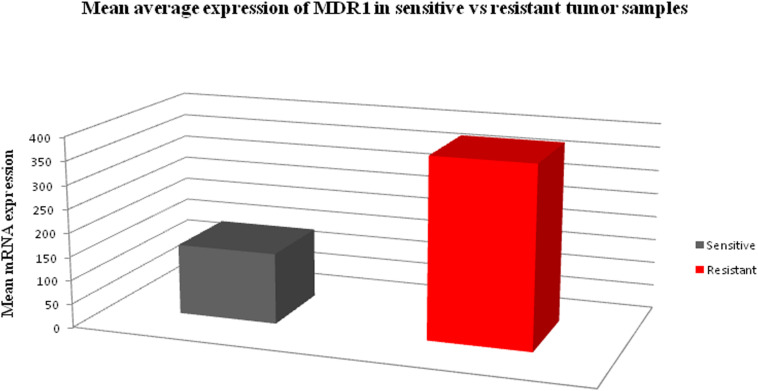
The bar chart diagram showing the comparative mean average expression between all resistant and sensitive ovarian tumor samples (380.14 in resistant vs. 149.47 in sensitive) respectively.

In order to determine the correlation between genotypes variants of MDR1 with mRNA expression to evaluate its relevance in terms of response to chemotherapy, prognosis and first we assessed the expression of mRNA levels in MDR1 gene of OC samples (both sensitive and resistant). The mRNA expression data of MDR1 were compared with relative expression results of SK-OV-3 and OVCAR-3 (ATCC ovarian cancer cell line) reported to be platinum resistant (Gao et al., 2014). When we compared the relative quantification data among tumor samples, we observed the highest expression relative to the OVCAR-3 of MDR1 gene in T-30, T-32, T-24, T-47, T-34, T-17, RT-31, T-48, T-36, and T-37 as shown in Figure 1. Notably, our qPCR results were matched with the reported finding of MDR1 gene expression in SK-OV-3, where RQ value has been shown to be nearly fivefold (Gao et al., 2014). Our comparison analysis between sensitive and resistant tumor samples showed that the majority of resistant samples (T-32, T-47, T-17, T-31, T-36, and T-37) have enhanced expression as compared to the sensitive samples. However, high expression was also observed in few sensitive samples (T-30, T-24, T-34, and T-48). Further, a comparison of mRNA expression level of MDR1 among CB/TX resistant tumor samples, showed a nearly 24.3 to 653-fold increase in expression level. However, one sample T-1 resistant to CB/TX plus GEMZAR showed the least mRNA expression level, i.e., 15-fold as compared to control OVCAR-3 Figure 1 and Table 6. Notably, we observed the second highest 493-fold expression of MDR1 in T-47 samples resistant to 5FU. Notably, our comparative analysis of mRNA expression between sensitive and resistant tumor samples revealed that the mean average expression of all the resistant tumor samples was much greater than those of the sensitive samples (380.14 in resistant vs. 149.47 in sensitive) respectively as shown in Figure 2.

**TABLE 6:** Stages of samples and their relationship with MDR Genotypes and mRNA expression with chemotherapeutic resistance profile.

Stage	Sample	Age	Genotype (Exon)			MDR Relative mRNA expression level	Chemotherapeutic resistant pattern
			12	21	26		
**III**	T1	61	C/T^μ^	GG^¥^	CC^¥^	15.0	CB/TX + GEMZAR
	T6	61	C/T^μ^	GG^¥^	C/T^μ^	24.3	CB/TX
	T27	61	C/T^μ^	GG^¥^	C/T^μ^	107.4	CB/TX
	T31	34	CC^¥^	T/T*	CC^¥^	271.1	CB/TX
	T36	33	C/T^μ^	T/T*	C/T^μ^	248.6	CB/TX
	T37	74	CC^¥^	GG^¥^	C/T^μ^	155.3	Carbopltin
**IV**	T17	57	T/T*	G/T^μ^	T/T*	271.5	5FU
	T32	43	T/T*	T/T*	T/T*	653.0	CB/TX
	T43	64	T/T*	GG^¥^	C/T^μ^	97.8	CB/TX
	T47	80	C/T^μ^	GG^¥^	C/T^μ^	493.0	5FU

¥, Wild Type; μ, Heterozygous mutation; *, Homozygous mutation; #, New SNP; CB, Carboplatin; TX, Paclitaxel; 5FU, Fluorouracil.

**FIGURE 3:**
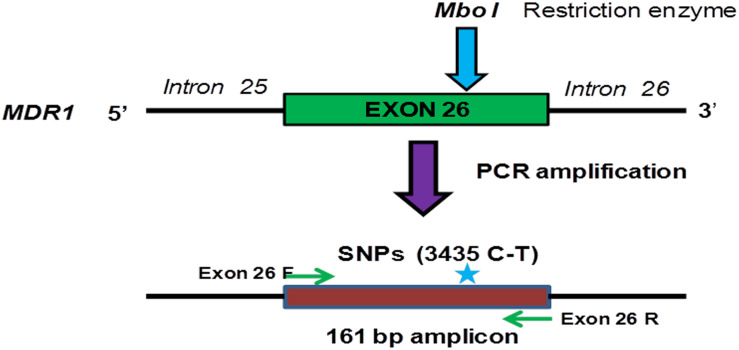
Schematic representation of PCR amplification and restriction digestion with *Mbo*I in order to determine the genotype of exon 26 (C3435T) of the *MDR1* gene in ovarian tumor samples.

### Genotyping of MDR 1 Gene

There is no information available regarding genotype frequency and its correlation with chemo resistance in ovarian cancer patients of Saudi ethnicity. In order to establish the correlation between MDR1gene expression and genotype with chemotherapeutic response to infer a clinical significance for the management of ovarian cancer patients, we have genotyped a total of 52 clinical ovarian tumor tissues along with 19 control samples of healthy women in exon-12 (C1236T), 21- (G2677T/A) and exon-26 (C3435T) of MDR1 gene, respectively. To genotype all the mentioned exon, we mostly used the Automated DNA sequencing method. However, exon-26 (C3435T) of MDR1 was genotyped by both RFLP as well as sequencing methods. The genotyping of exon 26 (C3435T) of MDR1 by RFLP analysis showed 35 heterozygous samples (C/T) at position C3435T on exon-26 of MDR1, whereas six samples were identified as homozygous mutant for TT allele (Supplementary Figure P1). Genotyping results of ovarian tumor samples and healthy controls are summarized in Table 7.

**TABLE 7:** Genotyping results of ovarian tumor and healthy control samples.

Genotyping results of DNA sequencing of ovarian tumor samples						
Gene	Exon	Wild Type	Heterozygous	Homozygous	Significance	Total
**MDR1**	12	CC (23) 44.2%	CT (20) 38.5%	TT (9) 17.33%	*P* = 0.03* *P* = 0.06^#^	52
	21	GG (35) 67.3%	GT (9) 17.3% GA (1) 1.9%	TT (7) 13.5%	*P* = 0.26* *P* = 0.2^#^	52
	26	CC (11) 21.2%	CT (35) 67.3%	TT (6) 11.5%	*P* = 0.003* *P* = 0.6^#^	52

**Genotyping results of DNA sequencing of control samples**						
**Gene**	**Exon**	**Wild Type**	**Heterozygous**	**Homozygous**	**Total**	

**MDR1**	12	CC (14) 73.7%	CT (1) 5.3%	TT (4) 21.0%	19	
	21	GG (10) 52.6%	GT (8) 42.1%	TT (1) 5.3%	19	
	26	CC (11) 57.9%	CT (6) 31.6%	TT (2) 10.5%	19	

Number of samples is mentioned in parenthesis. Significance value was calculated at *P* < 0.05. *, significance value of total mutations (heterozygous and homozygous) in ovarian tumor samples was calculated against healthy control women samples. #, significance value of homozygous and heterozygous mutations in ovarian tumor samples was calculated against healthy control women samples.

### Genotyping of Exon 12 (C1236T), 21 (G2677T/A), and 26 (C3435T) of MDR1 Gene by Automated DNA Sequencing

In order to correlate the mRNA expression of MDR1 genes involved in drug resistance in ovarian cancer with genotype variants, we carried out the genotyping analysis of exon 12 (C1236T), exon 21 (G2677T/A), and exon 26 (C3435T) of MDR1 gene in ovarian tumor tissues. The sequencing results were compared with a reported sequence using multiple alignment tools available at NCBI. The retrieved DNA sequence of each sample was also manually validated by observing the chromatograms as shown in Supplementary Figures P2A–C. Wild type and mutant alleles were identified as a single or heterozygous peak, respectively. In exon 12, 38.5% (20) were heterozygous mutants and 17.3% (9) were homozygous mutants. Our comparison analysis between ovarian tumor samples and healthy controls Table 7, we found a significant difference (*P* = 0.03) in exon-12. We further found a significant difference (*P* = 0.06) when compared between heterozygous and homozygous mutations. In exon-26, heterozygous mutations were found in 67.3% (35) samples, whereas 11.5% (6) samples were found to have homozygous mutation as shown in Table 7. The results of exon 26 genotypic differences were highly significant (*P* = 0.003) as compared to the controls Table 7. Notably, the heterozygous and homozygous mutations differences in exon 26 C3435T (*P* = 0.06) were as significant as the exon 12 C1236T. However, no significant differences (*P* = 0.26) were observed in exon 21 G2677T/A as compared to the control as shown in Table 7. The 17.3% (9) heterozygous mutation GT was observed as compared to the homozygous mutations TT (7) 13.5% without any significant differences (*P* = 0.2).

### Association Between Genotype Variants of Each Exon of MDR1 With Clinical Characteristics

The obtained results were statistically analyzed to observe any association among different histopathological features. In this respect, we applied Chi-Square test to analyze genotype variants of each exon with different tumor stages, grades, and histopathology as well as chemotherapy response. The analyzed results were not found to be statistically significant when genotypic variants of exons were compared with different stages of the ovarian tumor as Fisher’s exact test were more than 0.05 (*P* = 0.05). However, only genotypic variants of exon 12 were closely significant with a *p*-value of 0.06 (Table 8). Comparing the genotypic variants of exons with tumor grade, we observed no statistical significance as Fisher’s exact test values were more than 0.05 in each case (Table 9). Notably, a significant statistical association was observed between Exon 12 genotypic variants and histology of tumor tissue as *p*-value was found to be 0.028 (Table 10). Since the *p*-values for exons 21 and 26 were more than 0.05, therefore, we conclude that there have been no significant associations between tumor histology and genotypic variance among these exons (Table 10). Statistical analysis for genotypic variations in exons and response to chemotherapy revealed that there have been no associations between these two variables (Table 11). However, a significant association was observed in response to the chemotherapy with regard to the tumor stage (*P* = 0.021), though the response to chemotherapy was found to be non-significant with regard to the grade of the tumor (Table 12). The statistical difference in progression Free Survival (PFS) and Overall Survival (OS) among various characteristics of the ovarian tumor were analyzed by Mann–Whitney, and Jonckheere–Terpstra test as summarized in Supplementary Table S1. Notably, we observed a significant difference in PFS (*P* = 0.019^∗^) and OS (*p* = 0.047^∗^) between tumor grades 1 and 3. In other words, the patients in grade 3 lives shorter than patients having grade 1 or 2. Further, Mann–Whitney *U* test was also performed to calculate the difference in MDR1 exon’s (wild type vs. mutant) among PFS and OS. We did not observe any significant association between wild type and mutant MDR1 exon with PFS and OS in ovarian cancer patients (data not shown).

**TABLE 8:** Cross tabulation and chi-Square test for exon and stage.

Genotype		Stage				Fisher’s exact test
		I	II	III	IV	
Exon 12	Wild type	5	2	16	0	*P* = 0.060
	Mutant	2	2	19	6	
Exon 21	Wild type	5	2	25	3	*P* = 0.581
	Mutant	2	2	10	3	
Exon 26	Wild type	0	1	10	0	*P* = 0.196
	Mutant	7	3	25	6	

**TABLE 9:** Cross tabulation and chi-square test for exons and grade.

Genotype		Grade			Fisher’s exact test
		I	II	III	
Exon 12	Wild type	7	3	13	*P* = 0.921
	Mutant	7	4	18	
Exon 21	Wild type	11	4	20	*P* = 0.587
	Mutant	3	3	11	
Exon 26	Wild type	3	1	7	*P* = 1.000
	Mutant	11	6	24	

**TABLE 10:** Cross tabulation and chi-square test for exons and histology.

Genotype		Histology		Chi square test
		PS	NS	
Exon 12	Wild type	8	15	*P* = 0.028*
	Mutant	19	10	
Exon 21	Wild type	17	18	*P* = 0.488
	Mutant	10	7	
Exon 26	Wild type	5	6	*P* = 0.629
	Mutant	22	19	

*Significant at *P* ≤ 0.05.

**TABLE 11:** Cross tabulation and chi-Square test for exon and response.

Genotype		Response		Fisher’s exact test
		Sensitive	Resistance	
Exon 12	Wild type	17	2	*P* = 0.168
	Mutant	20	8	
Exon 21	Wild type	26	6	*P* = 0.704
	Mutant	11	4	
Exon 26	Wild type	8	2	*P* = 1.000
	Mutant	29	8	

**TABLE 12:** Cross tabulation and chi-square test for response with stage and grade.

Genotype		Response		Fisher’s Exact test
		Sensitive	Resistance	
**Stage**	I	5	0	*P* = 0.019*
	II	4	0	
	III	26	6	
	IV	2	4	

**Grade**	1	8	0	*P* = 0.203
	2	6	3	
	3	23	7	

*Significant at *P* < 0.05.

## Discussion

Epithelial Ovarian cancer represents the highest deaths amongst all gynecological cancers (Torre et al., 2018). In spite of the significant progress and advancements gained in the treatment modalities, yet 30% of advanced stage patients do not respond to standard chemotherapeutic regimen and most of the responders finally relapse over the time due to the emergence of multidrug resistance (MDR) mechanism (Cannistra et al., 2004; Auner et al., 2010; Guo et al., 2018). The genetic variations in ABCB1 gene seem to affect the pharmacokinetic as well as pharmacodynamics of many drugs, hence both together might lead to influence drug dose, and drug efficacy in terms of treatment response and adverse effects (McLean and Le Couteur, 2004; Hurria and Lichtman, 2007). In this perspective, three coding region SNPs variants, such as C1236T (exon 12, rs1128503), G2677T/A (exon 21, rs2032582) and C3435T (exon 26, rs1045642) of ABCB1/MDR1 gene polymorphisms have been well investigated and extensively characterized (Illmer et al., 2002; Kamazawa et al., 2002; Gréen et al., 2006). Multiple findings have confirmed that these SNPs variants are associated with altered mRNA levels, protein folding and drug pharmacokinetics (Cascorbi, 2006; Kimchi-Sarfaty et al., 2007; Longo et al., 2010). Further, association studies of SNPs C1236T, G2677T/A and C3435T in ABCB1 gene have been linked with altered mRNA expression and drug response in many cancers including OC (Gréen et al., 2006; Yin et al., 2009; Kim et al., 2017). However, data regarding the association of MDR1 genetic polymorphisms with mRNA expressions to correlate their prognostic significance for predicting chemotherapeutic response outcomes in Saudi ovarian cancer women are not available. In this perspective, our aim of the present study was to determine the correlation between SNPs of MDR1 and mRNA expressions in order to evaluate their relevance in terms of response to chemotherapy and prognosis. In this study, out of 52 OC patients, 47 cases were evaluated for chemotherapeutic response. The chemotherapeutic regimes selected by the oncologist at King Abdulaziz University Hospital, Jeddah are summarized in Table 5. The patients having recurrent, persistent, or progressive disease, the choices of chemotherapy have been adopted partly on the duration and type of response to initial therapy. For example, the platinum-sensitive disease, a platinum-based combination regimen (platinum/taxane) has been empirically chosen for therapy. For platinum-resistant disease oncologists empirically selected from an array of non-platinum regimens, mostly 5FU, FEMERA and gemcitabine (GEMZAR), all of which have been evaluated and proven to be clinically equivalent and acceptable for chemotherapy in such a pool of patients. Based on the above-mentioned criteria, the anti-cancer drug response evaluation results showed an over all 78.8% sensitive and 21.2% resistant patients respectively. Notably, our chemotherapeutic sensitivity data matches with other finding, where Trope and Kaern (2006) have also reported that around 20% Platinum-refractory patients do not respond to the platinum drugs and are considered as resistant and the remaining 80% are termed as a Platinum-sensitive as those cases responded better to platinum anti-cancer drugs at an early stage. Interestingly, all the chemoresistant OC tumor samples have also recorded in the disease recurrence group of patients having at stage III and IV and the highest resistant cases 70% (7/10) are recorded with platinum drug Carbo/Tax followed by non-platinum 5FU 66.7% (2/3) and GEMZAR 50% (1/2) respectively as shown in Tables 5, 6, 12. Our findings are very much consistent with other previous reports, where most of resistant tumors were also reported at stage III or IV and nearly, 30% ovarian cancer patients of this stage could not respond to treatment due to the development of MDR resistance (Ozols, 2006; Auner et al., 2010; Shuang et al., 2016). The high fold increase in the mRNA expression in nearly all resistant tumor along with few sensitive samples clearly suggest that high expression of MDR1 gene is not due to the specific chemotherapeutic drug as these tumor samples were resistant to either a combination of Paclitaxel and Carboplatin (CB/TX) or Carboplatin alone or Fluorouracil (5FU). It is well documented that Taxane (Paclitaxel) is a substrate of MDR1 not Carboplatin so, MDR1 most likely up-regulated under the prolonged exposure to Paclitaxel rather than Carboplatin. Notably, our observation regarding MDR1 expression in almost tumor samples, are very much supported by the previous study conducted in GROVCDDP Cisplatin-resistant ovarian cancer cell line, where Stordal et al. (2012) have shown that Cisplatin is not a substrate of a *P*-glycoprotein. They have demonstrated that over-expression of MDR1/*P*-gp is not due to specific response to a substrate (Cisplatin) rather, *P*-gp was up-regulated due to the common stress response (Stordal et al., 2012). Thus, we can suggest that MDR1 over-expression in both resistant (CB/TX or CB or 5FU) as well as in few sensitive OC tumor samples are not due to the specific chemotherapeutic drug response, rather the increased mRNA expression level is due to several stress response pathways thought to play a role in the regulation of *P*-gp expression (Kantharidis et al., 2000; Stordal et al., 2012). When we compared these resistant tumor samples with the genotype and mRNA expression profiles, we observed a common heterozygous mutation 1236 (C/T) in exon 12 of tumor samples of stage III. In contrast, homozygous mutations (T/T) were found to be at stage IV in most of the tumor samples. Similarly, in genotype variants of exon 26 (C3435T), the homozygous mutations (T/T) were observed at stage IV resistant tumor samples, suggesting the involvement of T allele in inducing the mRNA high expression and thereby contributing resistance phenotype in the late stage, such as III and IV. This remarkable observation may be justified by the facts that a homozygous mutation (TT) was identified in all the exon of T-32 resistant (CB/TX) tumor samples identified at stage IV, in which the highest mRNA expression has also been recorded as compared to other resistant cases as shown in Table 6. In general, our study observed increased mRNA expression of MDR1 in both heterozygous and homozygous mutations in exons 12 and 26 in majority of the resistant samples as shown in Table 6, and thus we can suggest that chemotherapeutic resistant development in tumor samples seem to be appeared not only due to combinatorial effect of more than one mutation but also the stage of tumor plays a significant role in conferring resistance phenotype in OC patients. We have also shown that the presence of T allele in exon 12 (C1236T) and especially exon 26 (C3435T) is associated with a greater increase in mRNA expression that eventually leads to confer poor response to chemotherapy. Remarkably, our finding is further substantiated with other studies in placenta and breast cancer, where Kafka et al. (2003) have shown an association of haplotype T with increased mRNA expression. However, our results were contrary to most other studies have failed to find an association between SNP (C3435T) and mRNA expression of MDR1 gene in OC (Hitzl et al., 2004; Gao et al., 2014). Notably, a recent study has shown an association between MDR1 mRNA expression and poor response to chemotherapy through the same study did not observe any relationship between genotype variants C3435T, G2677TA, and C1236T and mRNA expression (Doxani et al., 2013). Similarly, another study on Egyptian breast cancer patients also failed to establish any association between the MDR1 C1236T, G2677T/A polymorphisms and MDR1 mRNA expression (Fawzy et al., 2014). Further, an Australian study on ovarian cancer also did not find any association between SNPs in exon-12 (C1236T) and 21 (G2677T/A) and MDR1 expression (Gao et al., 2014). When we performed the Chi-Square Test, to analyze the genotype variants of each exon with different tumor stages, grades, and histopathology as well as chemotherapy response. We did not observe any significant association between genotypic variants of exons with different stages of the ovarian tumor as Fisher’s exact test were more than 0.05 (*p* ≥ 0.05). However, only genotypic variants of exon-12 were closely significant with *p*-value of 0.06 (Table 8). Similarly, no statistical significance observed, when genotypic variants of exons were compared with tumor grade as Fisher’s exact test values were more than 0.05 (Table 9). Notably, a statistically significant association was observed between Exon-12 genotypic variants and histology of tumor tissue as the *p*-value was found to be 0.028 (Table 10). Since the *p*-values for exons 21 and 26 were more than 0.05, therefore we conclude that there has been no significant association between tumor histology and genotypic variance among these exons (Table 10). Statistical analysis for genotypic variations in exons and response to chemotherapy revealed that there was no association between these two variables (Table 11). However, a significant association was observed in response to the chemotherapy with regard to the tumor stage (*P* = 0.019), though the response to chemotherapy was found to be non-significant with regard to the grade of tumor (Table 12). Notably, we observed a significant statistical difference in PFS (*P* = 0.019^∗^) and OS (*p* = 0.047^∗^) between tumor grades 1 and 3 but could not find any significant differences between other pairwise comparisons and hence we suggest that grade 3 ovarian tumor patients most likely live shorter than other grades like 1 or 2 (Supplementary Table S1). This could be further justified by our observation, where tumor stage II was found to possess the second highest values for PFS (14.5 (44) and highest OS 28.5 (57) median (IQR) months, respectively. Similarly, maximum PFS and OS were observed with tumor grade 1 with values 22.0 (35) months and 29.5 (39) months, respectively. Where as the grade 3 tumor found to show lower values for PFS and OS 8.0 (14) and 13.0 (22) median (IQR) months, respectively. Interestingly, based on histology, both PFS and OS were also found to be greater in ovarian tumor with serous histology than in the non-serous one (Supplementary Table S1). Thus, our observations suggest that a trend in decrease in the mean PFS and OS is associated with the advancement of tumor grade in Saudi ethnicities. Notably, our findings are very much supported by a large study conducted in the American population, where ovarian cancer patients with grade 3 tumor were found to be associated with decreased PFS and OS as compared to the tumor grade 1 (Winter et al., 2007). However, based on tumor histology, we found improved clinical outcomes in patients with papillary serous histology as compared to those with non-serous tumors (Supplementary Table S1) and surprisingly, our finding appeared to be contrary to results from American study (Winter et al., 2007).

Although, the present study does not find any association between wild type and mutant MDR1 exon with PFS and OS in Saudi ovarian cancer patients (data not shown). However, we observed wild type exons 12 (C1236T) and 21 (G2677T/A) of MDR1 more likely appeared to live longer than the mutant. Surprisingly, we found an inverse correlation with exon- 26 (C3435T), i.e., wild type less likely live longer than the mutant on ovarian cancer patients. Further, Mann–Whitney *U* test was also performed to calculate the difference in MDR1 exon’s (wild type vs. mutant) among PFS and OS. We did not observe any significant association between wild type and mutant MDR1 exon with PFS and OS in ovarian cancer patients.

Though, irrespective of the stages or mutation, most of the resistant tumor samples showed elevated MDR1 gene expression. Since more than one SNPs were identified in each resistant tumor samples, hence, we were unable to correlate with the individual SNPs variants impact on MDR1 gene expression and response to chemotherapy, however, the effect of subtle mutations of each exon on MDR1 gene expression cannot be ruled out. To validate individual SNPs effect on high expression of MDR1 gene in ovarian cancer, further research work will be needed to analyze the effect of these subtle mutations by *in vitro* transport and drug response assay.

## Conclusion

High level expression of mRNA of MDR1 correlates with chemotherapeutic resistance to combination chemotherapy in ovarian cancer. The exons 12 (C1236T) and exon 26 (C3435T) homozygous mutations (TT) seems to be very much associated with increased mRNA expression of MDR1 in resistant tumor patients. Hence, we can suggest that enhanced MDR1 expression level may be a useful prognostic significance to evaluate the drug response and prediction of new combinatorial chemotherapeutic agents in OC patients. Further, our findings revealed that inter individual variability in platinum based therapy might be anticipated by MDR1 genotypes and hence, genotypic profiling of ABCB1 gene along with non-P-gp ABC transporter genes, such as MRP1, MRP2, and MRP3 of individual OC patients might be a novel approach in terms of providing beneficial information for individualized chemotherapy. However, in order to determine the subtle effect of these MDR1 SNPs impact on protein expression as a prognostic significance, further research work will be essentially important to establish its individual mutation’s functional consequences on transporter efflux activity and chemotherapeutic response in ovarian cancer.

Moreover, the importance of non-P-gp transporter such as multidrug resistance-associated protein MRP1, MRP2, and MRP3 gene involvement with resistance to platinum-containing drugs in OC could not be ruled out. Hence, a better understanding of the mechanisms of chemotherapy resistance in OC patients, by increasing more knowledge about these genes such as MRP1, MRP2, and MRP3, whose genetic polymorphism may influence its protein expression which might in turn eventually lead to affect the outcome of chemotherapy in advanced ovarian cancer patients. Therefore, before reaching any definite conclusion with regard to the establishment of prognostic significance of MDR1 gene in predicting clinical correlation with response to chemotherapy and prognosis, more studies would be further needed based on larger samples, in order to get better insights into complete and distinct understanding on the ways to overcome drug resistance.

## Data Availability Statement

The raw data supporting the conclusions of this article will be made available by the authors, without undue reservation, to any qualified researcher.

## Ethics Statement

The studies involving human participants were reviewed and approved by the local Ethical Committee Board, King Abdulaziz University. Ethical clearance was also taken from King Fahd Medical Research Center, KAU. The patients/participants provided their written informed consent to participate in this study.

## Author Contributions

AH designed and supervised the study. QA, MA, and AW did the experimental work. KS and NA provided the samples and related clinical information. MR and AH wrote the manuscript and critically reviewed it.

## Conflict of Interest

The authors declare that the research was conducted in the absence of any commercial or financial relationships that could be construed as a potential conflict of interest.

## Footnotes

1 http://bioinfo.ut.ee/primer3-0.4.0/
