# Supplementary material for: MDR1 Gene Polymorphisms and Its Association With Expression as a Clinical Relevance in Terms of Response to Chemotherapy and Prognosis in Ovarian Cancer
Source: Front Genet. 2020 May 26;11:516. doi: 10.3389/fgene.2020.00516 (PMC7264409; doi:10.3389/fgene.2020.00516)
Supplement: Supplementary file 1 [file Presentation_1.pdf]

Figure P1 (A): RFLP analysis of MDR1 exon-26 (C3435T) on agarose gel

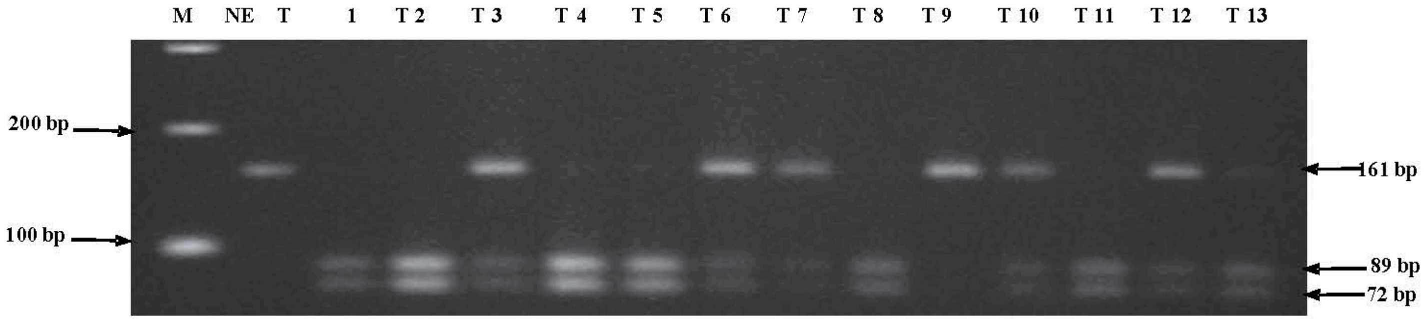

Figure P1 (A): 2.5% agarose gel showing restriction fragment length of the *MDR1*exon-26 (C3435T) genotype after restriction digestion with *MboI*. Lane 1 indicates 100bp marker , Lane 2 uncut, Lane 3 WT (C/C), Lane 4 WT (C/C), Lane 5 heterozygous (C/T), Lane 6 WT (C/C), Lane 7 WT (C/C), Lane 8 heterozygous (C/T), Lane 9 homozygous (T/T), Lane 10 WT (C/C), Lane 11 heterozygous (C/T), Lane 12 heterozygous (C/T), Lane 13 WT (C/C), Lane 14 heterozygous (C/T), Lane 15 heterozygous (C/T).

Figure P1 (B): RFLP analysis of MDR1 exon-26 (C3435T) on agarose gel

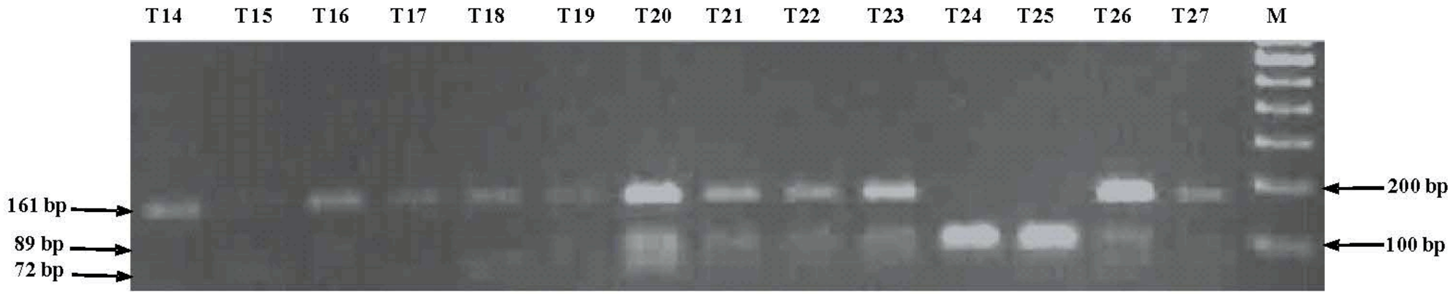

Figure P1 (B): 2.5% agarose gel showing restriction fragment length of the *MDR1* exon-26 (C3435T) genotype after restriction digestion with *MboI*. Lane 1-3 heterozygous (C/T), Lane 4 homozygous (T/T), Lane 5-12 heterozygous (C/T), Lane 13 WT (C/C), Lane 14 heterozygous (C/T), Lane 15 indicates 100bp marker.

Figure P1 (C): RFLP analysis of MDR1 exon-26 (C3435T) on agarose gel

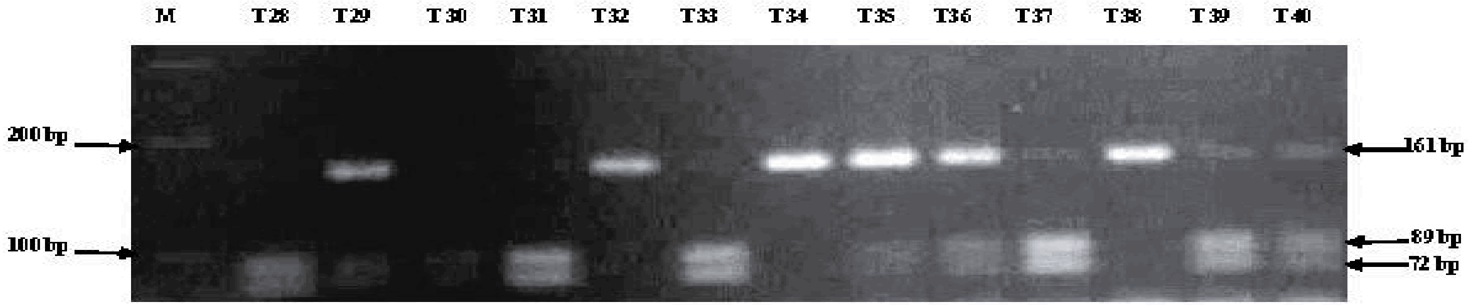

Figure P1 (C): 2.5% agarose gel showing restriction fragment length of the *MDR1* exon-26 (C3435T) genotype after restriction digestion with *Mbo I*. Lane 1 indicates 100bp marker, Lane 2-10 heterozygous (C/T), Lane 11 homozygous (T/T), Lane 12-13 heterozygous (C/T).

Figure P1 (D): RFLP analysis of MDR1 exon-26 (C3435T) on agarose gel

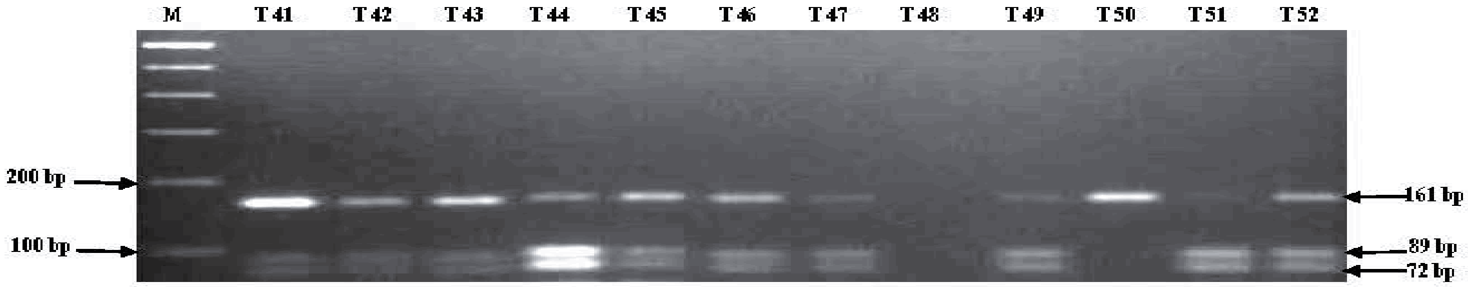

Figure P1 (D): 2.5% agarose gel showing restriction fragment length of the *MDR1* exon-26 (C3435T) genotype after restriction digestion with *Mbo I*. Lane 1 indicates 100bp marker, Lane 2-10 heterozygous (C/T), Lane 11 homozygous (T/T), Lane 12-13 heterozygous (C/T).

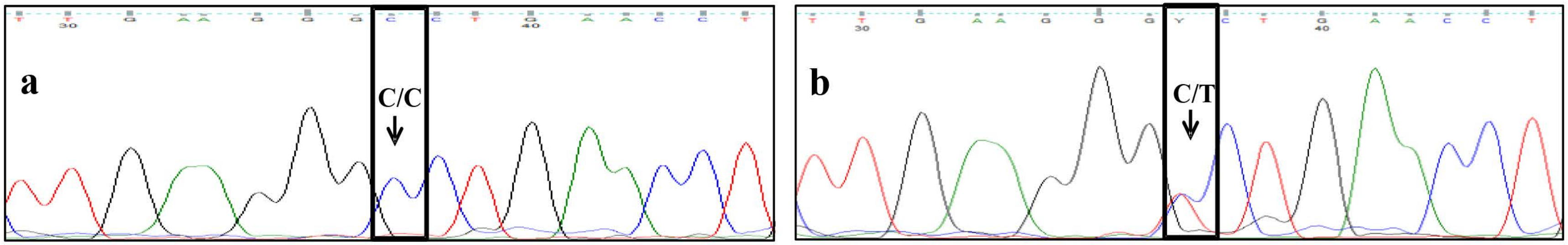

Figure P2 (A): Showing genotyping data of exon 12 (C1236T) of ABCB1 target sequence (a) wild type; (b) heterozygous mutant.

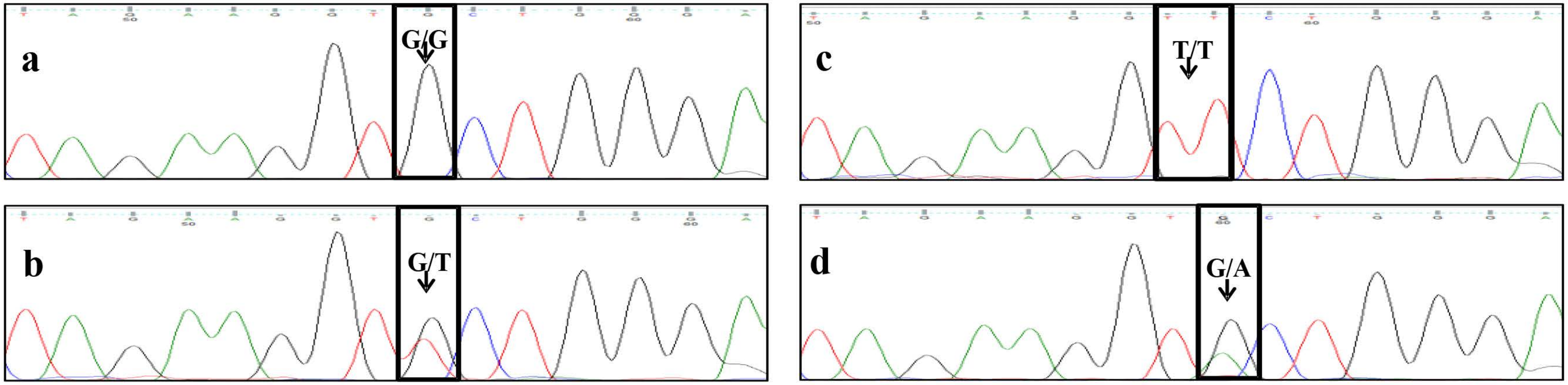

Figure P2 (B): Showing genotyping data of exon 21 (G2677T/A) of ABCB1 target sequence (a) wild type; (b) and (d) heterozygous mutant; (c) homozygous mutant.

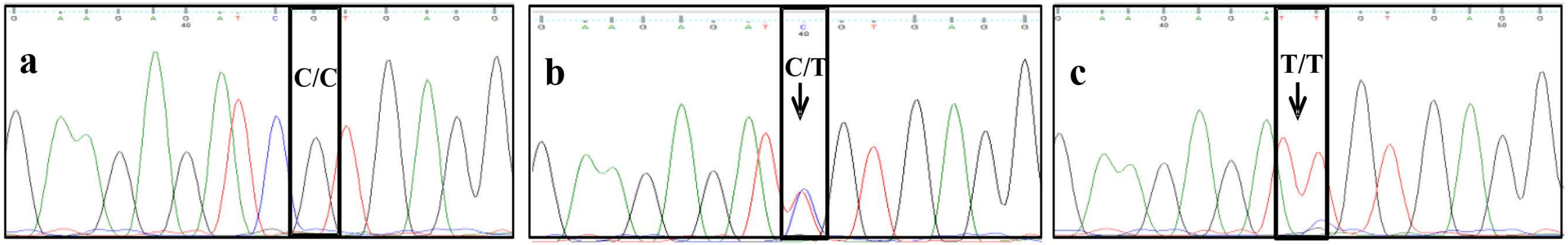

Figure P2 (C): Showing genotyping data of exon 26 (C3435T) of ABCB1 target sequence (a) wild type; (b) heterozygous mutant; (c) homozygous mutant.
