# Supplementary material for: MDR1 Gene Polymorphisms and Its Association With Expression as a Clinical Relevance in Terms of Response to Chemotherapy and Prognosis in Ovarian Cancer
Source: Front Genet. 2020 May 26;11:516. doi: 10.3389/fgene.2020.00516 (PMC7264409; doi:10.3389/fgene.2020.00516)
Supplement: Supplementary file 2 [file Table_1.docx]

**Supplementary Tables**

**Table S1:** Descriptive statistics (Median ± IQR), Mann Whitney, and Jonckheere–Terpstra test for ordered alternatives difference for PFS and OS among various characteristics of ovarian tumor

| **Tumor Characteristics** | **No. of patients** | **Survival** | | | |
| --- | --- | --- | --- | --- | --- |
|  |  | **PFS** | | **OS** | |
|  |  | **Median(IQR) (months)** | ***P value*** | **Median(IQR) (months)** | ***P value*** |
| Histology |  |  |  |  |  |
| PS | 27 | 12.0 (25) | *0.756* | 18.5 (29) | *0.420* |
| NS | 25 | 9.5 (22) |  | 11.5 (31) |  |
| Tumor Stage |  |  |  |  |  |
| I | 7 | 7.0 (6) | *0.089* | 7.0 (6) | *0.097* |
| II | 4 | 14.5 (44) |  | 28.5 (57) |  |
| III | 35 | 12.0 (26) |  | 15.0 (32) |  |
| IV | 6 | 24.5 (30) |  | 27.0 (21) |  |
| Tumor Grade |  |  |  |  |  |
| 1 | 14 | 22.0 (35) | *0.019******** | 29.5 (39) | *0.047****** |
| 2 | 7 | 11.0 (72) |  | 15.0 (72) |  |
| 3 | 31 | 8.0 (14) |  | 13.0 (22) |  |
| **Total** | **52** | **9.50 (23)** |  | **15 (29)** |  |

**PS = Papillary Serous; NS = Non-Serous; PFS= Progression- free survival; OS= Overall survival**
